# Nonlinear optical microscopy is a novel tool for the analysis of cutaneous alterations in pseudoxanthoma elasticum

**DOI:** 10.1007/s10103-020-03027-w

**Published:** 2020-05-06

**Authors:** Norbert Kiss, Luca Fésűs, Szabolcs Bozsányi, Flóra Szeri, Matthias Van Gils, Viktória Szabó, Anikó Ilona Nagy, Bernadett Hidvégi, Róbert Szipőcs, Ludovic Martin, Olivier Vanakker, Tamás Arányi, Béla Merkely, Norbert M. Wikonkál, Márta Medvecz

**Affiliations:** 1 Department of Dermatology, Venereology and Dermatooncology, Semmelweis University, 41 Mária Street, Budapest, H-1085 Hungary; 2 Wigner RCP, Institute for Solid State Physics and Optics, Hungarian Academy of Sciences, Budapest, Hungary; 3 The PXE International Center of Excellence in Research and Clinical Care, Department of Dermatology and Cutaneous Biology, Sidney Kimmel Medical College, Thomas Jefferson University, Philadelphia, PA USA; 4 Center for Medical Genetics, Ghent University Hospital, Ghent, Belgium; 5 Department of Biomolecular Medicine, Ghent University, Ghent, Belgium; 6 Department of Ophthalmology, Semmelweis University, Budapest, Hungary; 7 Heart and Vascular Center, Semmelweis University, Budapest, Hungary; 8 R&D Ultrafast Lasers Ltd, Budapest, Hungary; 9 PXE Reference Center (MAGEC Nord), Angers University Hospital, Angers, France; 10 Research Center for Natural Sciences, Institute of Enzymology, Hungarian Academy of Sciences, Budapest, Hungary

**Keywords:** Pseudoxanthoma elasticum, Nonlinear optical microscopy, Multiphoton microscopy, Elastin, Calcification

## Abstract

Pseudoxanthoma elasticum (PXE, OMIM 264800) is a rare autosomal recessive disorder with ectopic mineralization and fragmentation of elastin fibers. It is caused by mutations of the *ABCC6* gene that leads to decreased serum levels of inorganic pyrophosphate (PPi) anti-mineralization factor. The occurrence of severe complications among PXE patients highlights the importance of early diagnosis so that prompt multidisciplinary care can be provided to patients. We aimed to examine dermal connective tissue with nonlinear optical (NLO) techniques, as collagen emits second-harmonic generation (SHG) signal, while elastin can be excited by two-photon excitation fluorescence (TPF). We performed molecular genetic analysis, ophthalmological and cardiovascular assessment, plasma PPi measurement, conventional histopathological examination, and ex vivo SHG and TPF imaging in five patients with PXE and five age- and gender-matched healthy controls. Pathological mutations including one new variant were found in the *ABCC6* gene in all PXE patients and their plasma PPi level was significantly lower compared with controls. Degradation and mineralization of elastin fibers and extensive calcium deposition in the mid-dermis was visualized and quantified together with the alterations of the collagen structure in PXE. Our data suggests that NLO provides high-resolution imaging of the specific histopathological features of PXE-affected skin. In vivo NLO may be a promising tool in the assessment of PXE, promoting early diagnosis and follow-up.

## Introduction

Pseudoxanthoma elasticum (PXE, OMIM#264800) is a rare autosomal recessive connective tissue disorder characterized by ectopic mineralization and fragmentation of elastin fibers. At the genetic level, homozygous or compound heterozygous loss-of-function mutations in the *ABCC6* gene (OMIM#603234) are the main cause of PXE [1]. *ABCC6* encodes an ATP binding cassette protein which is crucial for connective tissue homeostasis. Loss-of-function of the ABCC6 protein results in reduced ATP release from hepatocytes. Since extracellular ATP is rapidly cleaved by ENPP1 to AMP and inorganic pyrophosphate (PPi), in the absence of ABCC6, PPi levels and pyrophosphate/phosphate ratio are decreased [2]. PPi is an anti-mineralization factor, thus low plasma levels of PPi likely contribute to aberrant ectopic mineralization. While low PPi levels unquestionably play a critical role in the pathogenesis of PXE, the underlying mechanism of the ABCC6-dependent ATP release has not yet been elucidated [2, 3]. Recently, it has been shown that plasma PPi levels are decreased in PXE and other patients with ectopic mineralization. This is a biochemical hallmark of the disease and it has been proposed that increasing the PPi level could be of therapeutic use [4].

Due to strong inter- and intrafamilial heterogeneity in phenotypic expression, the diagnosis of PXE may be challenging and often delayed. The prevalence of PXE is estimated to be between 1 in 25,000 and 100,000 [5], with women twice as likely to be affected [6]. Carrier frequency is appraised to be about 1 per 100 [7].

Typically, skin symptoms develop first during adolescence as yellowish papules appearing first on the neck, and later in flexural areas [6, 8]. Papules can coalesce into reticulated plaques resembling a cobblestone pattern and the skin becomes progressively slack, redundant, and inelastic. Sometimes, skin changes can be minimal or absent even in the presence of significant ocular or vascular manifestations [9]. However, more severe skin lesions tend to co-occur with more severe ophthalmological symptoms [10].

Ophthalmological manifestations usually appear several years after the onset of skin signs. The most common ocular findings in PXE are angioid streaks, which are disruptions of the choroidal Bruch’s membrane [11]. Drusen-like retinal pigment irregularities in the temporal part of the posterior pole called “peau d’orange” are often early findings [12]. Cardiovascular involvement is mainly characterized by arteriosclerosis through the calcification of the media that affect vessels of the extremities, coronary arteries, and the cerebral circulation [13].

Though PXE skin lesions can be identified clinically in most cases, skin biopsy and/or genetic analysis are required to confirm the diagnosis. Typical histologic features include short, fragmented, clumped, and calcified elastic fibers in the mid-dermis, but abnormalities of collagen fibers can also be observed [8, 9, 14].

However, the dermatologic diagnosis of PXE can be challenging due to its rarity, the often relatively late disease onset, and the phenotypic heterogeneity. Therefore, PXE is underdiagnosed and even if the diagnosis is established, it usually takes several years after the manifestation of the first symptoms [9, 15, 16]. In addition, PXE-like conditions, showing a broad clinical spectrum and genetic heterogeneity, have also been described, which should be considered in the differential diagnosis of PXE [17]. These include generalized arterial calcification of infancy-1 and infancy-2 (GACI1 and GACI2), caused by bi-allelic mutations in *ENPP1* or *ABCC6*, respectively [9], or the PXE-like syndrome with multiple coagulation factor deficiency, caused by bi-allelic *GGCX* mutations [18].

Nonlinear optical (NLO) microscopy is a novel non-invasive imaging technique which has been used to assess morphologic changes in a wide array of dermatological conditions, including skin cancer, metabolic disorders, and various genodermatoses [19–23]. NLO processes can be generated by ultrafast, picosecond or femtosecond, laser pulses providing stain-free, and submicron resolution imaging of the skin with deeper tissue penetration than reflectance confocal microscopy. Distinct components of the dermal connective tissue can be differentiated with NLO techniques. Collagen emits a strong second-harmonic generation (SHG) signal due to its non-centrosymmetric structure with high second-order nonlinear susceptibility, while elastin, among different endogenous chromophores, such as keratin and melanin, can be visualized by another NLO technique, two-photon excitation fluorescence (TPF) [24]. In addition, TPF was found to be suitable for the detection of calcium depositions in artery walls and to track calcified nodule growth in aortic valves ex vivo [25, 26]. In our present study, we visualized calcification and demonstrated the fragmentation of elastic fibers in the skin of PXE patients utilizing NLO microscopy imaging that allows the histologic diagnosis of the disease.

## Methods

### Patient data

Three female and two male PXE patients with a mean age of 53.8 ± 13.1 years were included in this study. All patients were diagnosed and managed at the Department of Dermatology, Venereology and Dermatooncology, the Department of Ophthalmology, and the Heart and Vascular Center of Semmelweis University, Budapest, Hungary. Best-corrected visual acuity measurement, near vision test, slit lamp examination, fundoscopy, and optical coherence tomography (OCT) were performed in all patients. All patients underwent cardiovascular evaluation which involved detailed history taking, physical examination, electrocardiography, a comprehensive echocardiographic examination, and carotid Doppler ultrasound. Patients’ data are summarized according to the Phenodex scoring proposed by Legrand et al. [27]. The study was approved by the local Ethics Committee in Budapest, Hungary (SE TUKEB no. 193/2017) with the requirement of written consent from all the participants.

### Plasma PPi measurements

Blood samples were collected from each patient and five age- and gender-matched healthy volunteers who served as controls. Platelet-free plasma was prepared, and PPi content was determined as described by Jansen et al. [2]. Briefly, the blood was drawn using a 22-gauge needle into CTAD containing BD Vacutainer® tubes (Ref: 367599, Becton, Dickinson and Company, Franklin Lakes, NJ, USA), supplemented with 50 μl of 15% K3 EDTA prior to sampling. The plasma fraction was separated by centrifugation at 1000*g* 4 °C for 10 min and transferred to platelet separation tubes (Centrisart I® 300.000 MW, 13279E, Sartorius, Göttingen, Germany). Platelet-free plasma was prepared at 2200 g for 30 min, 4 °C and stored at − 80 °C. PPi content of the samples was determined in an enzymatic way. First, PPi was converted to ATP in an assay containing 80 μM MgCl2, 50 mM HEPES pH 7.4, 32 mU/ml ATP sulfurylase (MO394L, New England Biolabs, Ipswich, MA, USA), and 16 μM adenosine 5′-phosphosulfate (A5508, Sigma-Aldrich, Saint Louis, MO, USA) by incubating samples/standards for 30 min at 37 °C followed by the inactivation of the enzyme at 90 °C for 10 min. In a consecutive step, ATP content was determined in a bioluminescent assay using 20 μl BacTiterGlo (G8230, Promega Madison, WI, USA) for 20-μl sample/standard. PPi concentration of plasma samples was calculated using calibration standards and corrected for initial plasma ATP concentrations.

### Molecular genetic testing

#### Molecular analysis of the ABCC6 gene

Genomic DNA was isolated from whole blood (QIAamp blood kit, Qiagen®, Hilden, Germany) and the coding region of the *ABCC6* gene was amplified using an established protocol. Primer sequences are available upon request. Direct sequencing was performed using an Applied Biosystems 3730xl Sequencer®, with ABI PRISM BigDye Terminator Cycle Sequencing Kit (Applied Biosystems®, Foster City, CA, USA). Nucleotide numbers are derived from gDNA *ABCC6* sequences (GenBank accession no. NM_001171).

### Multiplex ligation-dependent probe amplification analysis

MLPA analysis of the *ABCC6* gene was performed using the SALSA MLPA kit PO92-B3 (MRC-Holland, Amsterdam, The Netherlands) according to the manufacturer’s recommendations. MLPA fragments were detected using an ABI3130XL or ABI3730XL capillary electrophoresis system (Applied Biosystems, Foster City, CA, USA) and analyzed using Coffalyser (MRC Holland, Amsterdam, The Netherlands).

### Skin sample preparation and histopathology

Skin biopsies from PXE-affected not photoexposed areas of all patients and five age-matched healthy controls were collected, formalin-fixed, and paraffin-embedded. Skin sections were stained with hematoxylin and eosin (H&E), Weigert’s elastic (WE), von Kossa (VK), and Van Gieson (VG) stains. WE was used to stain elastic fibers, VK to reveal CaP minerals, while collagen was stained by VG. Histopathologic evaluation was performed by an expert dermatopathologist.

### NLO microscopy imaging and image processing

Separate deparaffinized, unstained sections from the same skin biopsies were prepared for NLO investigations. The utilized NLO imaging setup has been previously described [22, 23]. In brief, a FemtoRose 100TUN NoTouch tunable, femtosecond pulse Ti-sapphire laser (R&D Ultrafast Lasers Ltd., Budapest, Hungary) was operated at 800-nm wavelength, delivering ~ 190 fs pulses at a ~ 76-MHz repetition rate. To focus the laser beam, a 20× water immersion objective (W-Plan – APOCHROMAT 20×/1.0 DIC (UV) VIS-IR, Carl Zeiss, Jena, Germany) was employed. A commercial Axio Examiner LSM 7 MP laser scanning two-photon microscope system (Carl Zeiss, Jena, Germany) with custom-modified detection optics was used to capture images. TPF signal was separated with a 525/50-nm bandpass emission filter, while SHG was collected using a 405/20-nm filter. Mosaic images were captured from multiple field of views (FOV) with an individual imaging area of 420 × 420 μm^2^.

The acquired TPF and SHG images were merged and composed into two-channel mosaic images with ImageJ v1.46 software (NIH, Bethesda, MD, USA). In each sample, ten representative FOV were selected for further analyses. To determine the ratio of calcification, calcium deposits were outlined, their relative surface area was measured, and the number and length of elastin fibers were counted manually using ImageJ. CT-FIRE v.13 (LOCI, University of Wisconsin – Madison, WI, USA), a curvelet-based framework designed to analyze properties of collagen fibers was customized and run on the raw SHG images to calculate length and width for single collagen fibers [28].

### Statistical analysis

Unpaired, two-tailed Student’s *t* test, Mann-Whitney’s *U* test, and linear regression were used for statistical analysis using GraphPad Prism v6.0 software (GraphPad Software Inc., La Jolla, CA USA). *P* values less than 0.05 were considered statistically significant. The results are expressed as mean ± standard error.

## Results

**Fig. 1:**
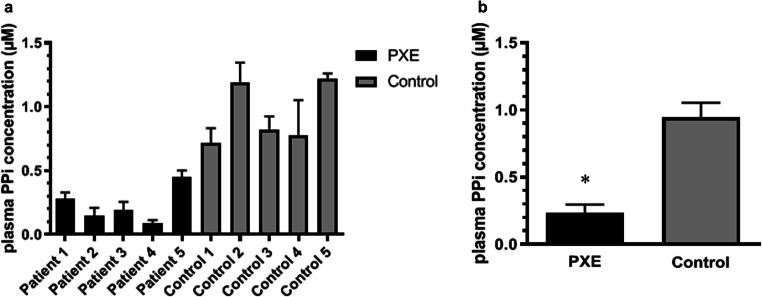
Plasma inorganic pyrophosphate (PPi) levels of our PXE patients compared with age- and gender-matched healthy controls. Blood samples were collected and platelet-free plasma was prepared. **a** Plasma PPi levels of all included patients were determined by first converting PPi to ATP, then measuring ATP content in a bioluminescent assay. PPi concentration of plasma was calculated based on calibration standards and corrected for initial plasma ATP concentration. **b** Plasma PPi levels in PXE vs control patients (mean ± SD 0.232 ± 0.063 μM vs 0.947 ± 0.108 μM) were compared using unpaired Student’s *t* test. *p < 0.05. Pt, patient; Co, control

Phenodex scores can be found in Table 1. Plasma PPi levels for PXE patients were significantly lower than for healthy controls (mean ± SD 0.232 ± 0.063 μM vs 0.947 ± 0.108 μM, respectively) (Fig. 1).

**Table 1:** Demographic data, Phenodex scores according to Legrand et al. [27], and molecular genetic data of the *ABCC6* gene in our PXE patients. Updated Phenodex score assesses six organ systems—skin (S), eye (E), gastrointestinal (G), vascular (V), cardiac (C), and renal (R)—to create phenotypic categories based on clinical findings

Pt. no.	Sex	Age (y)	Phenodex score	Allele 1		Allele 2	
				Gene	Protein	Gene	Protein
1	F	52	S2 E3 G0 V1 C0 R0	c.3421C>T^a^	p. R1141X^a^	c.1132C>T^a^	p.Q378X^a^
2	F	52	S2 E3 G0 V1 C1 R0	c.1552C>T^a^	p.R518X^a^	c.3662G>A^b^	p.R1221H^b^
3	M	34	S2 E2 G0 V0 C0 R0	c.105delA^c^	p.Val37Serfs*44^c^	c.3421C>T^a^	p.R1141X^a^
4	M	68	S3 E2 G0 V0 C0 R0	c.1944-1G>C^d^	-	exon 24-27del^c^	-
5	F	63	S3 E3 G0 V1 C0 R0	c.3421C>T^a^	p. R1141X^a^	c.1484T>A^b^	p.L495H^b^

*F*, female; *GI*, gastrointestinal; *M*, male; *Pt*, patient; *y*, years

^a^Nonsense mutation

^b^Missense mutation

^c^Deletion

^d^Splice-site mutation

### Molecular genetic testing

Bidirectional Sanger sequencing and MLPA analysis of *ABCC6* confirmed the diagnosis of PXE in all patients (Table 1). In patients 1, 2, 3, and 5, compound heterozygous bi-allelic pathogenic variants were identified. Moreover, in patient 4, an exon deletion and a novel splice-site mutation, c.1944-1G>C, have been detected. In silico mutational analysis was carried out using Human Splicing Finder v3.1 software (Aix Marseille Université, Marseille, France) in order to estimate the impact of the novel variant. The c.1944-1G>C change was predicted to fully disrupt the wild-type acceptor site, underscoring the pathogenicity of this mutation [29].

### Histopathology

**Fig. 2:**
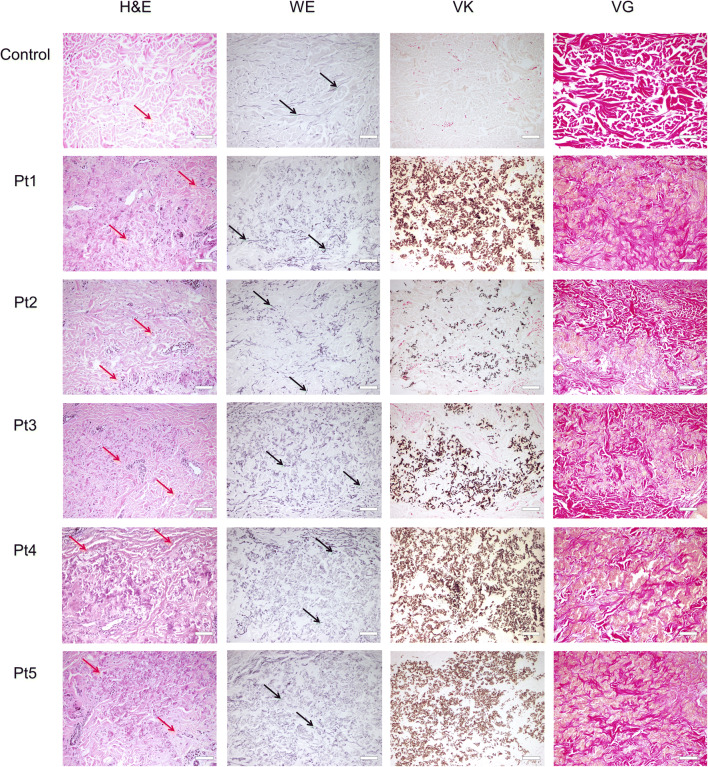
Representative histology images of the mid-dermis of healthy and PXE-affected skin, stained with hematoxylin and eosin (H&E), Weigert’s elastic (WE), von Kossa (VK), and Van Gieson’s (VG) staining. Red arrows, fibroblasts; black arrows, elastic fibers. Scale bars display 200 μm

The papillary dermis and the deep layers of the dermis appeared to be unaffected in all patients. However, the mid-dermis of PXE patients exhibited prominent changes. H&E staining showed irregularly shaped, clumped, and faintly basophilic elastic fibers as well as numerous fibroblasts (Fig. 2). WE staining revealed polymorphic, fragmented, and mineralized elastic fibers. VK staining displayed mid-dermal salt deposits and clumps of calcified elastin fibers, which were absent in the healthy samples. Finally, VG staining revealed disrupted, abnormal collagen fibers surrounding abundant mineral deposits, compared with dense, interwoven collagen structure in controls.

### NLO microscopy imaging

**Fig. 3:**
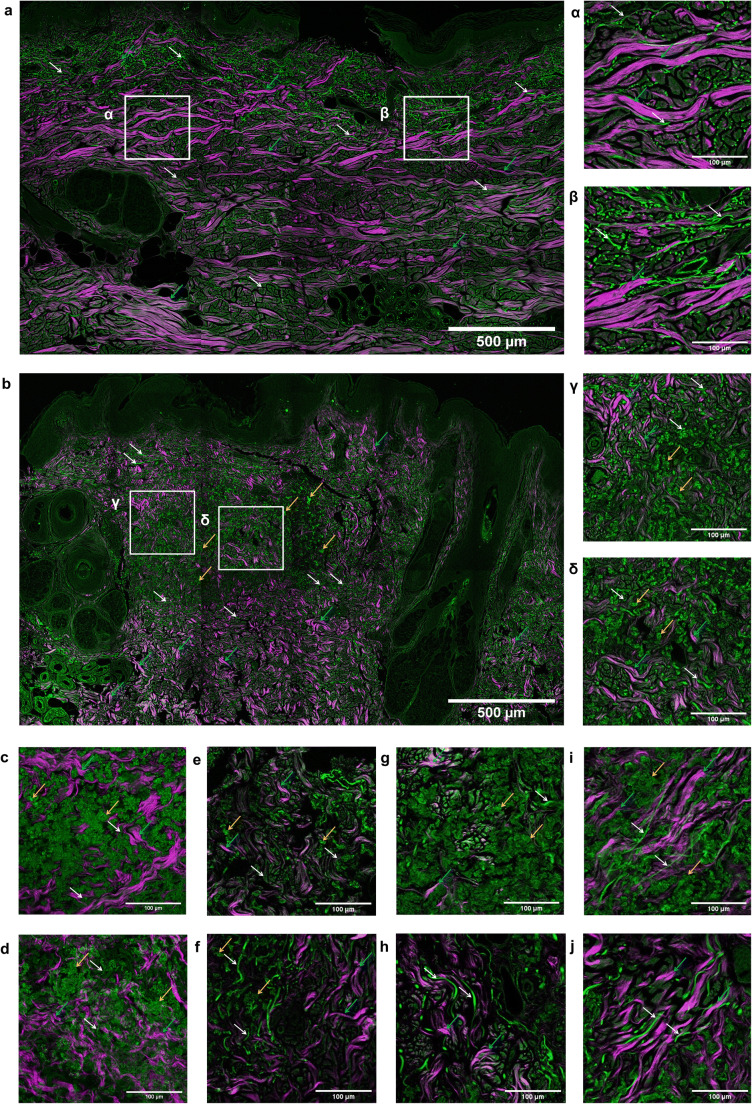
Two-photon excitation fluorescence (TPF) and second-harmonic generation (SHG) images of PXE-affected and healthy skin. TPF signal (shown in green color) is mainly emitted by elastin in the dermis, while SHG (magenta) displays the collagen structure, both generated at 800-nm excitation wavelength. 420 × 420 μm^2^ frames were captured and assembled into (**a**, **b**) mosaic images of skin samples with 300 × 300 μm^2^ high zoom images of the dermis (α, β, γ, δ). **a** Control skin, **b** skin of patient 3. High zoom inserts were marked with (**c**–**j**). Representative high zoom images of the mid-dermis, **c**, **d** patient 1, **e**, **f** patient 2, **g**, **h** patient 4, **i**, **j** patient 5. White arrows, elastin fibers; blue arrows, collagen fibers; yellow arrows, calcium deposits. Scale bars display 500 μm for mosaic images and 100 μm for high zoom images

**Fig. 4:**
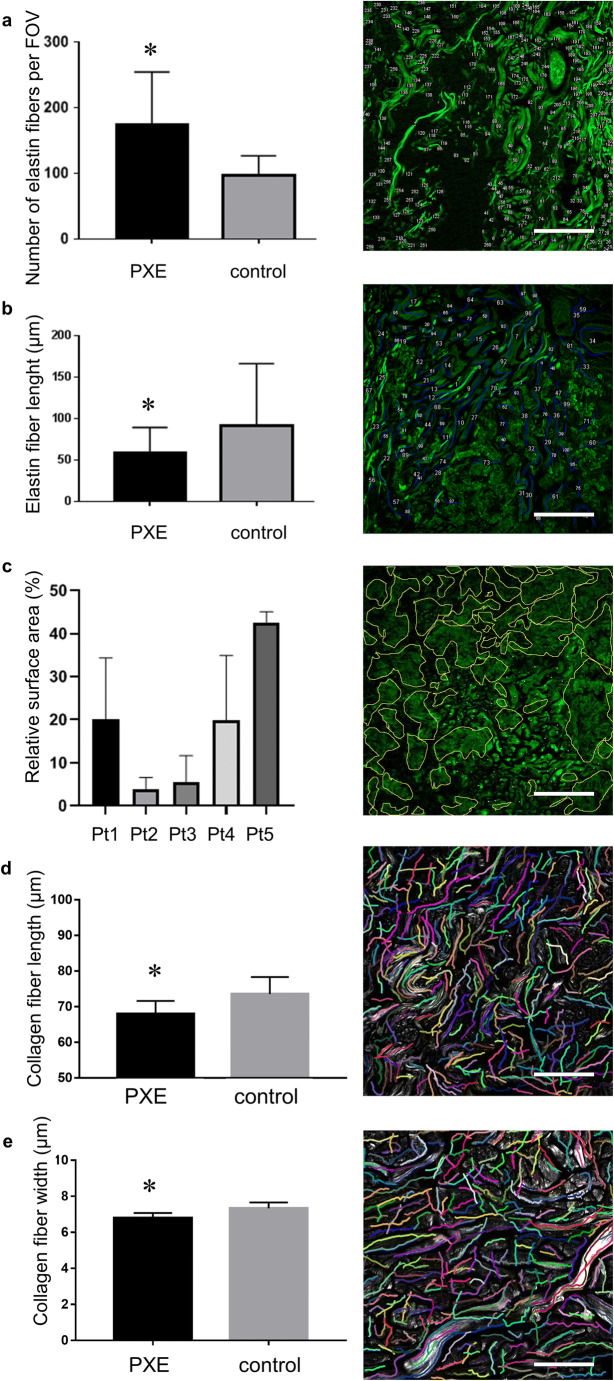
Extent of calcification and amount of elastin fibers in two-photon excitation fluorescence (TPF) images of the dermis of PXE patients and healthy, and results of CT-FIRE analysis of second-harmonic generation (SHG) images of collagen in the dermis of PXE-affected and control skin. **a** Number of elastin fibers in PXE vs control patients (mean ± SD 175.7 ± 79.49 μm vs 106.8 ± 23.92 μm), **b** length of elastin fibers in PXE vs control patients (59.49 ± 29.9 μm vs 91.64 ± 74.72 μm), **c** relative calcification surface area in each PXE patient. Right panels show representative TPF images of PXE for each parameter. Statistical analyses were carried out using Mann-Whitney *U* test. **e**, **f** Collagen fiber length (mean ± SD 67.99 ± 3.612 μm vs 73.53 ± 4.782 μm) (**e**) and width (6.797 ± 0.274 μm vs 7.327 ± 0.325 μm) were compared using Student’s *t* test after customized CT-FIRE analysis of raw SHG images. Representative images of CT-FIRE v.13 (LOCI, University of Wisconsin – Madison, WI, USA) analysis of patient 1 and a healthy control. Scale bars display 100 μm. *p < 0.05

NLO microscopic images of healthy and PXE-affected skin samples are displayed in Fig. 3. In the mid-dermis of the control samples, the TPF channels showed a network of wavy, branching elastic fibers, while in the SHG channel, randomly organized collagen bundles in a basket weave-like pattern were observed. Conversely, the mid-dermis of PXE patients comprised of spacious calcified areas and fragmented, clumped elastin fibers as revealed by the TPF channel. When we counted the number of elastin fibers (indicated with numbering in the right-side panel of Fig. 4a) in PXE patients, a significant increase has been found compared with healthy controls (175.7 ± 79.49 vs 97.56 ± 29.30). Elastin fiber length (outlined in blue in Fig. 4b) was significantly lower in PXE (59.49 ± 0.66 μm vs 91.64 ± 5.6 μm). Measuring the calcium deposit relative surface area (marked in yellow in Fig. 4c), we found significant calcification to be present in each patient but also that the general extent of calcification varied considerably among patients. No calcification was found in the controls. We also assessed if the extent of calcification correlated with plasma PPi levels and found no association (data not shown). SHG images from PXE patients showed sparsely distributed, irregular, and sometimes coiled collagen fibers (Fig. 3). With the use of CT-FIRE algorithm, we found significantly shorter collagen fibers (67.99 ± 0.79 μm vs 73.53 ± 1.04 μm) and decreased fiber width (6.8 ± 0.06 μm vs 7.33 ± 0.07 μm) in the PXE samples compared with controls (Fig. 4d–e). Similar to the histopathological analysis, the papillary dermis and deep dermis were morphologically normal for all samples.

## Discussion

The occurrence of severe complications among PXE patients highlights the importance of early diagnosis so that prompt multidisciplinary care can be provided to patients. Our results confirm previous data that suggested that PXE patients have PPi levels lower than 50% of the average healthy individual [2, 30]. However, the range of PPi levels is wide, both in patients and healthy individuals that complicate the interpretation of PPi levels. Furthermore, variability between measurements from different research laboratories suggests a need for better standardization of the technique before assessing the applicability PPi test in PXE.

In our cohort of 5 patients, we found the most frequent mutation p.R1141X with an allele frequency of 0.3, which is similar to what has been reported previously for the European population [9]. Most of the other mutations we identified are also frequent in this population, such as the large deletion of ex 24-27 or the Q378X, which was reported to be a result of gene conversion with *ABCC6* pseudogene 1 [31, 32]. We report here a novel splice-site mutation as well. In addition, we described here a detailed protocol for the *ABCC6* genetic analysis in an attempt of standardization.

Generally, PXE is first suspected by a trained dermatologist who notices the subtle clinical signs of the disease. Molecular genetic testing for *ABCC6* is a key step in the diagnosis of PXE, but due to the low prevalence of PXE, its use is often limited to the final confirmation of diagnosis [33]. While there are various techniques for ophthalmologic and cardiovascular assessment, currently, conventional histopathological examination is the sole widely applied objective method for the diagnosis of skin lesions in PXE [8]. Unfortunately, the accurate evaluation requires a skilled dermatopathologist [34, 35], and the effectiveness and specificity of VK and elastic fiber staining for the detection of calcification are often disputed. Therefore, alternative approaches to the histopathological diagnosis are highly needed.

Here, we used NLO to determine whether this technique is able to detect characteristic changes seen in PXE. We sought to visualize calcification and to perform a novel numeric analysis of the changes of connective tissue fibers in the skin of the investigated patients utilizing NLO microscopy imaging. We were able to show the degradation of elastin fibers in the samples of our PXE patients that confirm findings in previous histopathologic reports. In addition, we were able to visualize the mineralization of elastin fibers as also a hallmark feature of the disease. We showed here that the extensive mid-dermal calcium deposition harbors endogenous fluorescent properties. In addition, we introduced a numeric method to quantify changes in number and length of elastin fibers and the extent of calcification, as the two could be clearly distinguished based on their morphology in the captured submicronic resolution TPF images. The increase of number of elastin fiber count in PXE patients is the result of fiber fragmentation that is a consequence of aberrant mineralization [14]. The lack of correlation between plasma PPi levels and the extent of calcification may be due to the small cohort size. We also detected significant abnormalities in the SHG images of collagen fibers. Impaired collagen fibrillogenesis is a previously described feature of PXE, although it was considered highly aspecific [14]. Given that previous findings of the collagen morphology in PXE were based on conventional histopathology staining and SHG shows notably higher specificity to collagen, previous findings may need to be revisited using SHG [36].

The unique previous report that used NLO imaging in PXE analyzed a skin sample from a single PXE patient [37]. They found the alteration of elastin fibers; however, the NLO setup used in their study was not capable of visualizing calcium deposits, an important feature of PXE-affected skin. Based on our findings, NLO seems to enable high-detail visualization of calcium deposits and—more importantly—the alterations of elastin fibers, the specific features of PXE-affected skin at early stages. Thus, our setup provides a great improvement of the technique and emphasizes the potential of NLO in the diagnostics of PXE. NLO analysis revealed a complex pattern of mid-dermal alterations in PXE patients. This opens new clinical perspectives in the diagnosis and follow-up of PXE patients. Although in the present study we applied label-free NLO microscopy imaging on ex vivo skin samples, handheld devices capable of NLO microscopy imaging could be used for in vivo diagnostics [38]. Further studies are needed to establish the sensitivity and specificity of NLO approaches and their place in the diagnostic workup of PXE. Indeed, NLO techniques are promising tools to provide non-invasive “optical biopsies” of the skin in various disorders and could replace conventional histopathology which would decrease the disease burden for patients. We propose this method to be used as an important tool for early and non-invasive diagnosis of PXE, prior to confirmation by genetic tests. Also, to date, non-invasive biomarkers for the monitoring of treatment response in PXE are not available, and serial skin biopsies would not be feasible as scarring induces calcification. As various therapeutic options have been proposed recently for PXE [3], non-invasive monitoring of efficacy could be an important application of NLO in the future. Finally, one should note that in vivo NLO could also be applicable for the detection, characterization, or monitoring of other skin conditions with cutaneous calcinosis.

## Data Availability

The authors confirm that the data supporting the findings of this study are available within the article. Code availability: Software: ImageJ version 1.46 RRID:SCR_003070; CT-FIRE version 13 (LOCI, University of Wisconsin – Madison, WI, USA) is available at the following link: https://loci.wisc.edu/software/ctfire.
